# Curative effects of fucoidan on acetic acid induced ulcerative colitis in rats via modulating aryl hydrocarbon receptor and phosphodiesterase-4

**DOI:** 10.1186/s12906-022-03680-4

**Published:** 2022-07-23

**Authors:** Alaa Bagalagel, Reem Diri, Ahmad Noor, Deina Almasri, Hussain T. Bakhsh, Hussam I. Kutbi, Mohammed M. H. Al-Gayyar

**Affiliations:** 1 Department of Clinical Pharmacy, Faculty of Pharmacy, King Abdulaziz University, Jeddah, Saudi Arabia; 2 Department of Biochemistry, Faculty of Pharmacy, Mansoura University, 35516 Mansoura, Egypt; 3 Department of Pharmaceutical Chemistry, Faculty of Pharmacy, University of Tabuk, 71491 Tabuk, Saudi Arabia

**Keywords:** Aryl hydrocarbon receptor (AhR), Cyclic adenosine monophosphate (cAMP), Heme oxygenase-1 (HO-1), Interleukin (IL)-22, Nuclear factor erythroid 2-related factor 2 (Nrf2), Phosphodiesterase-4 (PDE4), Ulcerative colitis

## Abstract

**Background:**

Ulcerative colitis (UC) is an inflammatory bowel disease. Fucoidan, sulfated polysaccharide of brown seaweed, demonstrates various pharmacological actions as anti-inflammatory, anti-tumor and anti-bacterial effects. Therefore, we opt to investigate the potential curative effects of fucoidan in experimentally induced UC in rats through modulating aryl hydrocarbon receptor (AhR), phosphodiesterase-4 (PDE4), nuclear factor erythroid 2-related factor 2 (Nrf2) and Heme Oxygenase-1 (HO-1).

**Methods:**

UC was induced in rats using intracolonic 2 ml of 4% acetic acid. Some rats were treated with 150 mg/kg fucoidan. Samples of colon were used to investigate gene and protein expression of AhR, PDE4, Nrf2, HO-1 and cyclic adenosine monophosphate (cAMP). Sections of colon were stained with hematoxylin/eosin, Alcian blue or immune-stained with anti-PDE4 antibodies.

**Results:**

Investigation of hematoxylin/eosin stained micro-images of UC rats revealed damaged intestinal glands, severe hemorrhage and inflammatory cell infiltration, while sections stained with Alcian Blue revealed damaged and almost absent intestinal glands. UC results in elevated gene and protein expression of PDE4 associated with reduced gene and protein expression of AhR, IL-22, cAMP, Nrf2 and HO-1. Finally, UC increased the oxidative stress and reduced antioxidant activity in colon tissues. All morphological changes as well as gene and protein expressions were ameliorated by fucoidan.

**Conclusion:**

Fucoidan could treat UC induced in rats. It restored the normal weight and length of colon associated with morphological improvement as found by examining sections stained with hematoxylin/eosin and Alcian Blue. The curative effects could be explained by enhancing antioxidant activity, reducing the expression of PDE4 and increasing the expression of AhR, IL-22 and cAMP.

## Introduction

Ulcerative colitis (UC) is one of the inflammatory bowel disorders that attacks the mucosal lining of colon. Active UC is characterized by many clinical manifestations as bloody diarrhea, bowel urgency, abdominal pain, weight loss, fever and malaise [1]. The major problem associated with UC is bowel urgency that is considered a bothersome disruptive symptoms and affects more than of 80% of UC patients [2]. UC takes place as a result of chronic inflammation on smooth muscle tone and increased contractile responses in the rectum [3].

Although, UC takes place at any age, it commonly influences young age population leading to negative impact on quality of life associated with economic burden [4]. Epidemiology of UC is extremely elevated over the last decade and affects 8.8-23.1/100,000 person in North America, 0.6-24.3/100,000 person in Europe, and 7.3-17.4/100,000 person in Oceania [5].

The mucosal layer of the gastrointestinal tract is usually open to millions of antigens that arises from many sources as food, environment and microbiome. It is physically protected and separated by a thick layer of mucin covering the epithelium layer [6]. UC results in a damage of the epithelium with subsequent elevation in the permeability of mucosa, leading to increased uptake of the antigens and increased stimulation of gut immune system [7].

Aryl hydrocarbon receptor (AhR) is a xenobiotic receptor and a ligand-dependent transcription factor. It is located inside cytoplasm as inactive compound. After binding of the ligand, AhR complex moves into nucleus leading to expression of target genes [8]. Patients with UC have reduced activity of AhR [9]. Activation of AhR is associated with production of IL-22, enhancing epithelial barrier function, changing gut microbiota, enhancing enzymes metabolism, inhibiting immune response, and boosting mucosal healing [10].

For thousands of years, many people all over the world based on traditional medicines to cure several diseases because of their better availability, lower side effects and reduced cost. One of these natural compounds is fucoidan, which is sulfated polysaccharide of brown seaweed. It demonstrates various pharmacological actions such as anti-inflammatory, anti-tumor and anti-bacterial effects [11]. Moreover, it has been investigated in preclinical studies for immunomodulatory, anti-oxidant, anti-angiogenic, anti-viral and anti-hyperglycemic activities [12, 13]. It was used in treating UC through affecting colonic pathology, cytokine gene expression and Enterobacteriaceae [14, 15]. However, no previous study explored the curative effects of fucoidan against UC through modulating AhR. We used intrarectal administration of acetic acid to induce colitis in rats, which is considered a significant model of experimental colitis. It induces inflammation and ulceration in rectum and colon of rats [16]. Therefore, we aimed to conduct the following research to investigate the potential therapeutic effects of fucoidan in experimentally induced UC in rats through investigating its effect on AhR, phosphodiesterase-4 (PDE4), nuclear factor erythroid 2-related factor 2 (Nrf2) and Heme Oxygenase-1 (HO-1).

## Methods

### Animals and treatment outlines

The research was conducted on forty-eight Sprague Dawely rats (180-200 g). Rats were kept in standard conditions of temperature (23-25 °C) and humidity (50-60%) with regular 12 h light/12 h dark cycle. All methods were carried out in accordance with guidelines and regulations of working with experimental animals and the work protocol was approved by the local ethical committee in Faculty of Pharmacy, Mansoura University, Egypt. All methods are reported in accordance with ARRIVE guidelines (https://arriveguidelines.org) for the reporting of animal experiments. Rats were divided into four groups with twelve rats each:

#### Control group

Under ether light anesthetized, rats were treated with a soft pediatric lubricated catheter and underwent intracolonic 2 ml saline. Rats were kept horizontally to prevent draining of saline. Rats were kept free access to food and water without any treatment during the experiments.

#### Control group treated with fucoidan

The rats were treated exactly as the control group then treated with 150 mg/kg fucoidan (Sigma Aldrich Chemicals Co., St Louise, MO, US) by oral gavage daily for 2 weeks.

#### UC group

Under ether light anesthetized, rats were treated with a soft pediatric lubricated catheter and underwent intracolonic 2 ml of 4% acetic acid. Rats were kept horizontally to prevent draining of acetic acid [17].

#### UC treated with fucoidan

After induction of UC in rats, they were given 150 mg/kg fucoidan by oral gavage daily for two weeks.

Only one previous study illustrated the role of fucoidan in treating UC in mice using two doses: a high dose of 300 mg/kg and a low dose of 100 mg/kg [18]. In addition, there was no previous study used fucoidan in treating UC in rats. Therefore, a set of preliminary studies were performed testing four different concentrations of fucoidan, 100 mg/kg, 150 mg/kg, 200 mg/kg and 250 mg/kg. The dose of 150 mg/kg is selected as it was found to be the lowest dose with curative effects.

### Sample collection

After animal sacrifice, the whole colon was separated, measured and weighed. Part of colon was cut followed by fixation in 10% buffered formalin for morphologic and immunohistochemical analysis. Another part of the colon was separated and homogenized in a 10-fold volume of ice-cold sodium potassium phosphate buffer (0.01 M, pH 7.4) containing 1.15% KCl. The supernatant was stored at − 80 °C.

### Morphologic analysis

After separation of colon, it was cut into five-micrometer sections. The colon sections were stained with either hematoxylin/eosin or Alcian Blue stain. The mitotic figures were calculated from observing sections stained with hematoxylin/eosin by examining ten fields by high power in each rat.

### Immunohistochemistry

Five-micrometer thick paraffin sections were cut from a paraffin block of colon tissues. Sections were immune-stained with phosphodiesterase-4 (PDE4) antibodies (MyBioSource, Inc. San Diego, CA, USA) at 4 °C. Sections were then incubated with horseradish peroxidase conjugate antibody. Finally, sections were counterstained with hematoxylin and observed under microscope in a masked manner. The intensity of immune staining was evaluated using the score 0, no positive cells per high power field, 1 for infrequent, small number positive cells, 2 for common, moderate number of positive cells, and 3 for widespread, high numbers of positive cells.

### Measurement of oxidative stress and antioxidant activities

Colon lysate levels of malondialdehyde (MDA, Cat no. MD 25 29), reduced glutathione (Cat no. GR 25 11) and glutathione peroxidase (Cat no. GP 2524) were assessed using commercially available kits (BioDiagnostic Company, Giza, Egypt). Measurement of MDA depends on the interaction with thiobarbituric acid in acidic medium at 95 °C for 30 min to form the pink thiobarbituric acid reactive product, which can be measured at 534 nm. Assessment of reduced glutathione is based on the reduction with DTNB to produce a yellow compound, which can be measured at 405 nm. Activity of glutathione peroxidase is indirectly measure by oxidation of NADPH to NADP^+^, which is accompanied by a decrease in absorbance at 340 nm. Peroxynitrite was quantified using the method of Beckman [19]. In brief, peroxynitrite mediated nitration of phenol to form nitrophenol, which was measured at 412 nm.

### Enzyme-linked immunosorbent (ELISA) assay

Commercially available ELISA kits were used for assessment of aryl hydrocarbon receptor (AhR, Cat no. MBS726370), PDE4 (Cat no. MBS725350), cyclic adenosine monophosphate (cAMP, Cat no. MBS160960), nuclear factor erythroid 2-related factor 2 (Nrf2, Cat no. MBS752046), Heme Oxygenase-1 (HO-1, Cat no. MBS2508238) and IL-22 (Cat no. MBS2515891) (MyBioSource, Inc. San Diego, CA, USA) according to manufacturer’s instructions.

### Quantitative real-time polymerase chain reaction (RT-PCR)

The gene expression of AhR, IL-22, PDE4, cAMP, Nrf2 and HO-1 mRNA in rat colon was performed as described previously by our group [20, 21]. β-actin was used as a housekeeping gene and internal reference control. The gene specific PCR primers used were summarized in Table 1.

**Table 1:** The primers set used for detection of gene expression in rats

Gene symbol	Primer sequence from 5′- 3′	Gene bank accession number
β-actin	F: TCCGTCGCCGGTCCACACCC R: TCACCAACTGGGACGATATG	NM_031144.3
AhR	F: TCCCGTGTCTTTCAGCTGTC R: GCTCGGACTCTGAAACTTGC	AM902286.1
PDE4	F: AAGTAGCCGAATGGCAGCTC R: AGGCTAGTGTGGAGGCCATA	NM_001167806.2
cAMP	F: CCACGTCCTACATCCTCGTT R: AAGTGGTAGGGGCACCTTCT	AF053980
Nrf2	F: CTCTCTGGAGACGGCCATGACT R: CTGGGCTGGGGACAGTGGTAGT	NM_031789
HO-1	F: CACCAGCCACACAGCACTAC R: CACCCACCCCTCAAAAGACA	NM_012580
IL-22	F: CAACCGCACCTTTATGCTGG R: ATCCTTGGTTTGACTCCTCG	NM_001191988.1

### Statistical analysis

Data were presented as mean ± standard deviation. For evaluation of normality of sample distribution, Kolmogorov–Smirnov (K–S) test was used. One-way analysis of variance (ANOVA) was used to compare between groups followed by post hoc Bonferroni correction test. Statistical analyses were done using SPSS version 20 (Chicago, IL, USA). Statistical significance was predefined as *P* ≤ 0.05.

## Results

### Effect of fucoidan on UC-induced alteration in colon length and weight

**Fig. 1:**
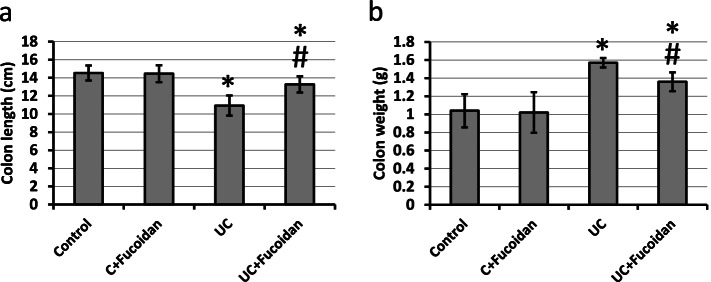
Effect of ulcerative colitis (UC) and 150 mg/kg fucoidan on colon length (**a**) and (**b**) weight. * Significant difference as compared with control groups at *p <* 0.05. # Significant difference as compared with UC group at *p <* 0.05

UC results in a significant reduction in colon length associated with significant elevation in the colon weight as compared with the length and weight of control groups. Treatment of UC rats with fucoidan significantly reversed these effects in UC group with affecting the control group (Fig. 1).

### Effect of fucoidan on UC -induced morphological changes

**Fig. 2:**
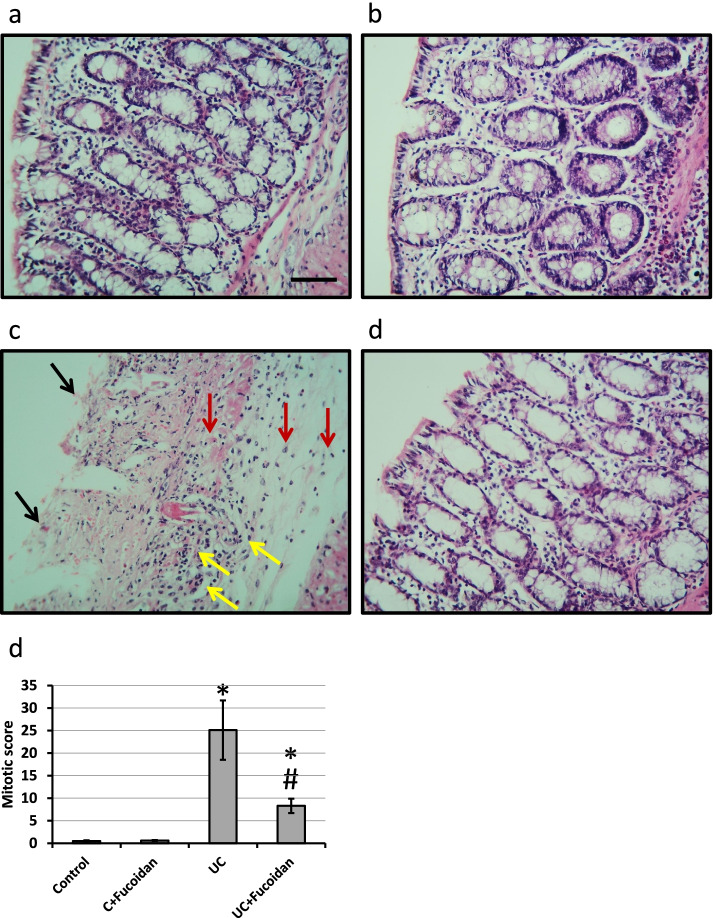
Colon sections stained with hematoxylin and eosin in control group (**a**), control treated with 150 mg/kg fucoidan (**b**), ulcerative colitis (UC, **c**) and UC treated with 150 mg/kg fucoidan (**d**). Mitotic figure was determined in 10 fields of high field power and expressed as mean ± standard deviation (**e**). Black arrows represented damaged intestinal glands, yellow arrows represented severe hemorrhage and red arrows represented inflammatory cell infiltration in the mucosa and the submucosa. * Significant difference as compared with control groups at *p <* 0.05. # Significant difference as compared with UC group at *p <* 0.05. Scale bar is 50 μm

Microscopic images of colon sections of control groups stained with hematoxylin/eosin showed normal intestinal glands. The images of colon sections in the UC group showed damaged intestinal glands, infiltration of inflammatory cell in mucosa and submucosa and severe hemorrhage, Treatment of UC with fucoidan revealed some improvement in intestinal cell structure with significant improvement in mitotic score (Fig. 2).

**Fig. 3:**
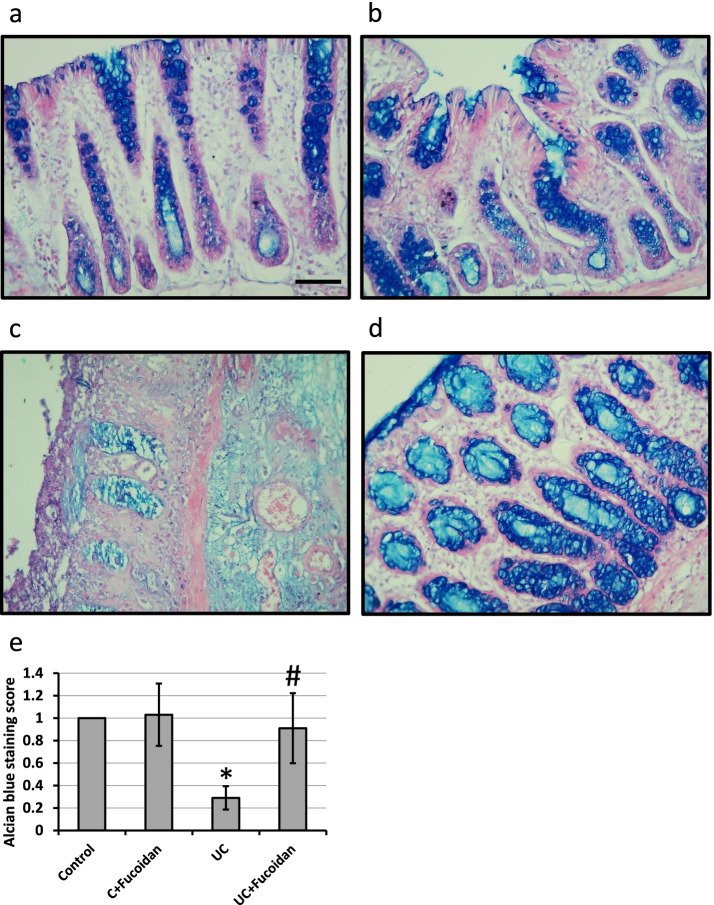
Colon sections stained with Alcian Blue stain in control group with the normal bluish stained intestinal glands (**a**), control treated with 150 mg/kg fucoidan with normal intestinal glands (**b**), ulcerative colitis (UC) with damaged and almost absent intestinal glands (**c**) and UC treated with 150 mg/kg fucoidan with less marked affection of the glands (**d**) as well as score of Alcian blue staining (**e**). * Significant difference as compared with controls group at *p <* 0.05. # Significant difference as compared with UC group at *p <* 0.05

Microscopic images of colon sections stained with Alcian Blue in control rats showing normal bluish stained intestinal glands. Examination of sections of UC group revealed damaged and almost absent intestinal glands. The sections of UC rats treated with fucoidan showed less marked affection of glands compared to UC group (Fig. 3).

### Effect of fucoidan on UC-induced reduction in expression of AhR

**Fig. 4:**
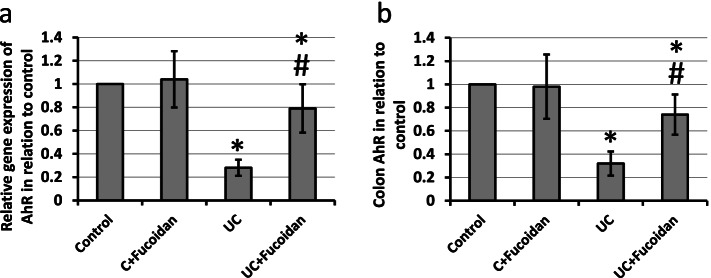
Effect of ulcerative colitis (UC) and 150 mg/kg fucoidan on gene (**a**) and protein expression (**b**) of aryl hydrocarbon receptors (AhR). * Significant difference as compared with control groups at *p <* 0.05. # Significant difference as compared with UC group at *p <* 0.05

We checked the effect of fucoidan on gene and protein expression of AhR. UC resulted in 72 and 68% reduction in gene and protein expression of AhR, respectively, as compared with the control groups. Treatment of UC rats with fucoidan significantly attenuated both gene and protein expression of AhR as compared with UC group without affecting the control group (Fig. 4).

### Effect of fucoidan on UC-induced decrease in expression of IL-22

**Fig. 5:**
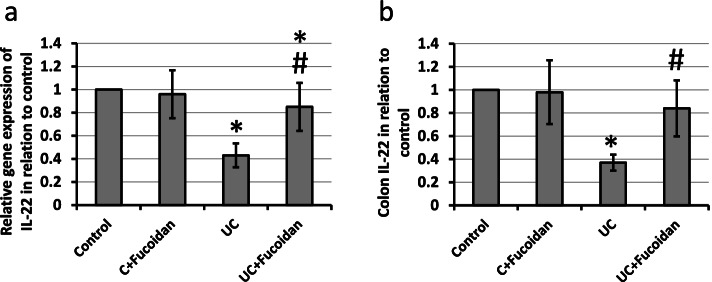
Effect of ulcerative colitis (UC) and 150 mg/kg fucoidan on gene (**a**) and protein expression (**b**) of IL-22. * Significant difference as compared with control groups at *p <* 0.05. # Significant difference as compared with UC group at *p <* 0.05

IL-22 is an anti-inflammatory cytokine that is reduced in UC. We checked the effect of fucoidan on gene and protein expression of IL-22. UC resulted in 57 and 63% reduction in gene and protein expression of IL-22, respectively, as compared with control groups. Treatment of UC rats with fucoidan significantly attenuated both gene and protein expression of IL-22 as compared with UC group without affecting the control group (Fig. 5).

### Effect of fucoidan on UC-induced expression of PDE4

**Fig. 6:**
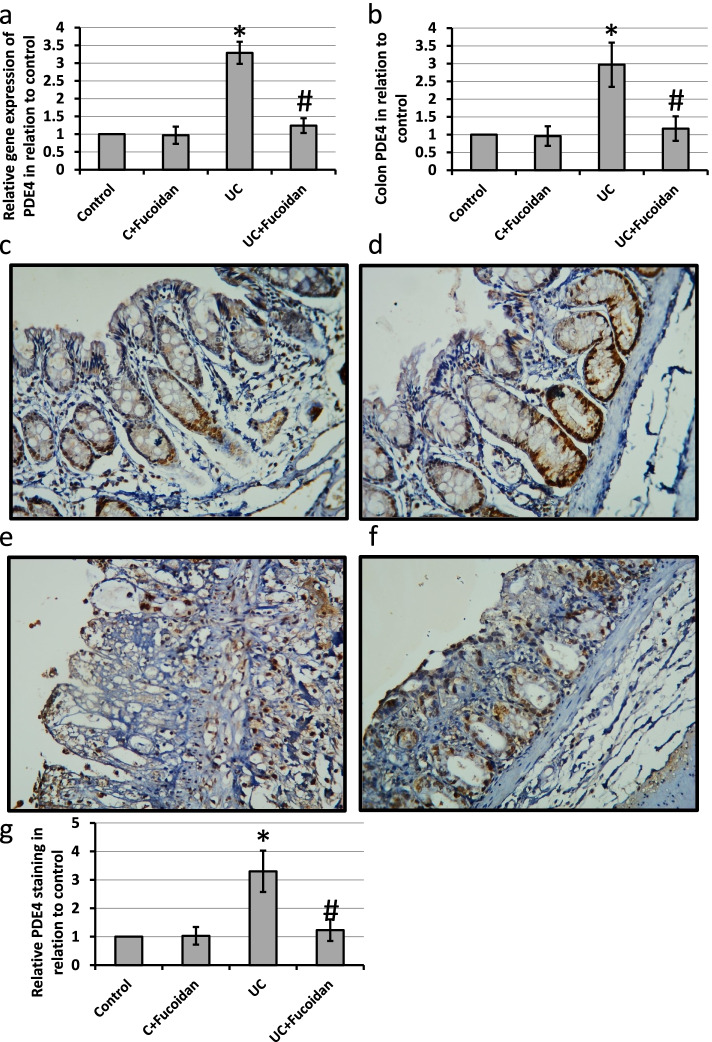
Effect of ulcerative colitis (UC) and 150 mg/kg fucoidan on gene expression of phosphodiesterase-4 (PDE4, **a**) and its protein level (**b**) as well colon sections stained with anti-PDE4 antibodies in control group (**c**), control group treated with fucoidan (**d**), UC group (**e**) and UC group treated with fucoidan (**f**) as well as immunohistochemistry score of positive staining (**g**). * Significant difference as compared with control group at *p <* 0.05. # Significant difference as compared with UC group at *p <* 0.05

UC caused 3.29- and 2.97-fold increase in the gene and protein expression of PDE4, respectively as compared with the control groups. In parallel, investigation of colon sections stained with anti-PDE4 antibodies revealed intense reaction and immune staining of the colon tissues in UC group. Treatment of UC rats with fucoidan significantly reduced the expression of PDE4 compared with UC group associated with reduction in the immune staining of colon sections stained with anti-PDE4 antibodies (Fig. 6).

### Effect of fucoidan on UC-induced reduction in expression of cAMP

**Fig. 7:**
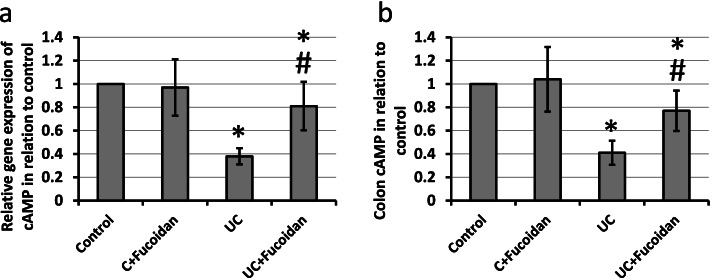
Effect of ulcerative colitis (UC) and 150 mg/kg fucoidan on gene (**a**) and protein expression (**b**) of cyclic adenosine monophosphate (cAMP). * Significant difference as compared with control groups at *p <* 0.05. # Significant difference as compared with UC group at *p <* 0.05

UC resulted in 62 and 59% reduction in gene and protein expression of cAMP, respectively, as compared with the control groups. Treatment of UC rats with fucoidan significantly attenuated both gene and protein expression of cAMP as compared with UC group (Fig. 7).

### Effect of fucoidan on UC-induced decreased expression of both Nrf2 and HO-1

**Fig. 8:**
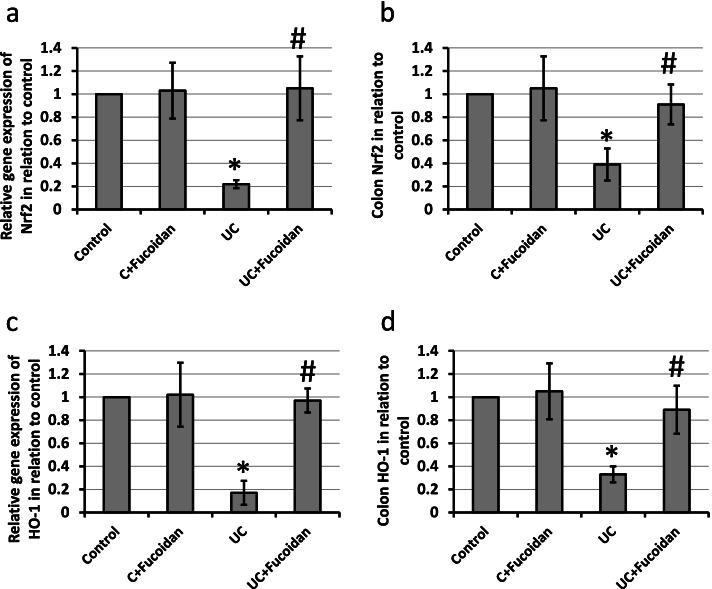
Effect of ulcerative colitis (UC) and 150 mg/kg fucoidan on gene expression of Nrf2 (**a**) and HO-1 (**c**) as well as protein levels of Nrf2 (**b**) and HO-1 (**d**). * Significant difference as compared with control groups at *p <* 0.05. # Significant difference as compared with UC group at *p <* 0.05

Induction of UC in rats leads to 78 and 83% reduction in the gene expression of Nrf2 and HO-1, respectively, associated with 61 and 67% reduction in protein levels of Nrf2 and HO-1, respectively, as compared with the control groups. Treatment of UC rats with fucoidan reversed these effects in UC group without affecting the control group (Fig. 8).

### Effect of fucoidan on UC-induced activation of oxidative stress

**Fig. 9:**
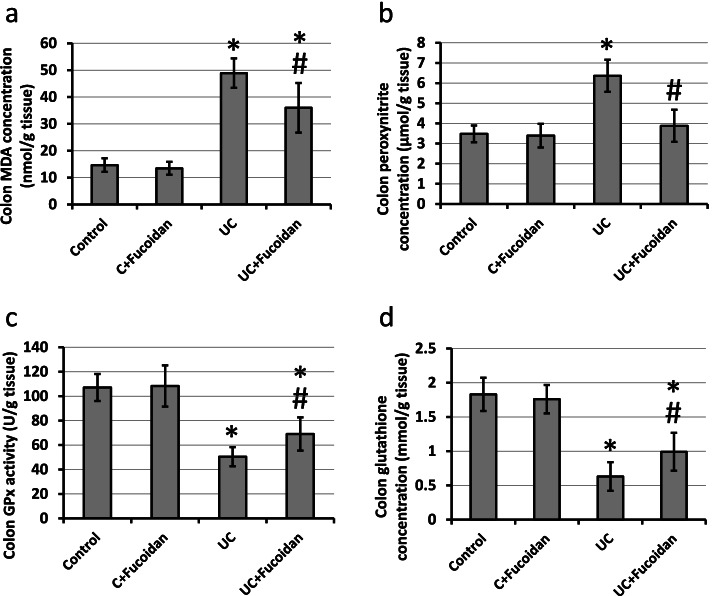
Effect of ulcerative colitis (UC) and 150 mg/kg fucoidan on malondialdehyde (MDA, **a**), peroxynitrite (**b**), glutathione peroxidase (GPx, **c**) and reduced glutathione (**d**). * Significant difference as compared with control groups at *p <* 0.05. # Significant difference as compared with UC group at *p <* 0.05

UC caused significant increase in the colon concentration of MDA and peroxynitrite associate with significant reduction in the activity of GPx and concentration of reduced glutathione as compared with the control rats. Treatment of UC rats with fucoidan blocked these effects in UC group without affecting the control group (Fig. 9).

## Discussion

Induction of UC in rats using acetic acid results in a significant reduction in colon length associated with a significant increase in the colon weight. In addition, investigation of colon sections stained with hematoxylin/eosin revealed damaged intestinal glands, severe hemorrhage and infiltration of inflammatory cell in the mucosa and submucosa associated with extreme elevation in mitotic score. Moreover, examination of colon sections stained with Alcian Blue revealed damaged and almost absent intestinal glands. Treatment of rats with fucoidan significantly restored normal length and weight of colon as well as restoration of normal shape of mucosa, submucosa and intestinal glands, indicating curative effects of fucoidan against UC. Fucoidan is used previously in treating UC through affecting colonic pathology, cytokine gene expression and Enterobacteriaceae [14, 15]. However, this the first time to report the ability of fucoidan to produce its protective effects in UC through affecting AhR, PDE4, cAMP, Nrf2 and HO-1.

Inside gastrointestinal tract, epithelium possesses a noticeable ability to self-renewal leading to replenishment of epithelial cells every 3 to 4 days. The mature epithelial cells could efficiently regenerate after any intestinal injury [22]. In the resting state, AhR inside cytoplasm is linked to a heat shock protein. When the receptor is bind to ligand, AhR detach from the shock proteins and transfer inside nucleus and reacts with genes containing xenobiotic responsive elements [23]. AhR responds to any endogenous ligands that came from the dietary and microbiota metabolites. Therefore, deficiency or alteration in AhR pathway are linked to elevated inflammatory responses especially in gut environment. It is significantly reduced in UC leading to inactivation of epithelial barrier, alteration of gut microbiota and overexpression of proinflammatory mediators [10]. We found that induction of UC in rats caused about 72% reduction in expression of AhR in rats that was reversed by treating with fucoidan without affecting the control group. However no previous study illustrated the role of fucoidan in increasing the expression of AhR.

We next investigated the role of IL-22 in UC. It is a new cytokine that was discovered in year 2000. It is found to be only expressed in non-hematopoietic cells such as hepatocytes, intestinal and respiratory epithelial cells. Therefore, it particularly aims innate immune responses without any direct effect on adaptive immune cells [10]. In addition, IL-22 enhanced the formation of a firm inner mucus layer to prevent bacterial invasion. It works through promoting the production of functional Muc1 and glycosylation [24]. Moreover, it could enhance mucosal healing which was proved by many experimental approaches such as mucosal healing after epithelial damage induced by dextran sulfate sodium [25] making IL-22 target for many therapies. Finally, IL-22 exhibits anti-inflammatory effects in UC [26]. We found that induction of UC in rats results in reduction in the expression of IL-22 that was attenuated by treating with fucoidan. However no previous study linked fucoidan treatment with expression of IL-22.

PDE4 is a target in many inflammatory disorders, such as chronic obstructive pulmonary disease, allergic dermatitis, psoriasis and psoriatic arthritis [27]. Activation of PDE4 resulted in overproduction of proinflammatory cytokines and chemokines leading to subsequent activation and infiltration of immune cells inside inflamed tissues. Thus, inhibitors of PDE4 have dramatic therapeutic activities in treating UC symptoms [28]. Inhibition of PDE4 is linked to increase in intracellular concentrations of cAMP, a critical downregulatory signal. cAMP inhibits the production of IFN-*γ*, TNF-*α* and IL-17 and associated with increased production of IL-10 [29]. However, we found that treating rats with fucoidan reversed UC-induced elevation in the expression of PDE4 associated with reduction in expression of cAMP. No previous study illustrated the ability of fucoidan to modulate PDE4 expression. However, fucoidan was previously reported to affect the expression of cAMP in diabetes in vivo and in vitro [30] and in amyloid beta1-42-induced signaling in glial cells and transfected HEK293 cells [31], but no previous study illustrated the effect of fucoidan on cAMP in UC.

Nrf2 is a remarkable antioxidant transcription factor. Under oxidative stress, Nrf2 is activated and transfer inside nucleus, and elevated the expression of downstream antioxidant enzymes making Nrf2 as a critical player in activity of antioxidant system [32]. Overexpression of Nrf2 was reported to improve UC [33]. Therefore, drugs that could activate Nrf2 are expected to have therapeutic potential against UC. One of the downstream of Nrf-2 is HO-1 [34]. HO-1 is an antioxidant protein that constitutes a defense network against oxidative stress damage and prevents colon tissue oxidative damage [35, 36]. We found that treatment with fucoidan significantly elevated the expression of Nrf2 and HO-1 associated with reduction in the concentration of MDA and peroxynitrite and increased GPx and reduced glutathione. Fucoidan was reported previously to increase expression of both Nrf2 and HO-1 in chronic kidney disease in mice [37], cyclophosphamide-induced liver and kidney injury in mice [38], diabetes-induced renal fibrosis in vivo and in vitro [39] and LPS-induced acute lung injury in mice [40]. However, no previous study illustrated the effect of fucoidan on UC.

## Conclusion

Fucoidan significantly treat experimentally induced UC in rats. It improves the morphological structure of the colon cells as indicated by examining sections stained with hematoxylin/eosin and Alcian Blue. Fucoidan ameliorated UC-induced increase in the expression of PDE4 as well as UC-induced reduction in the expression of AhR, IL-22, cAMP, Nrf2 and HO-1 leading to activation of antioxidant and anti-inflammatory systems and protective mechanisms inside the colon. We believe that our results can be readily translated to clinical use. Fucoidan is a highly safe natural product with LD50 value of 2000 mg/kg for oral use compared with 150 mg/kg in our study. In addition, fucoidan is administered orally in our study which represented protection by eating seaweed. Finally, the contents of fucoidan ranged from 4 to 33% of the weight of seaweeds. Therefore, the dose used during this study could be easily obtained by consuming seaweeds.

## Data Availability

The datasets used and/or analysed during the current study are available from the corresponding author on reasonable request.

## Abbreviations

AhR: Aryl hydrocarbon receptor
cAMP: Cyclic adenosine monophosphate
HO-1: Heme Oxygenase-1
Nrf2: Nuclear factor erythroid 2-related factor 2
PDE4: Phosphodiesterase-4 (PDE4)
UC: Ulcerative colitis
